# BDNF Alleviates Neuroinflammation in the Hippocampus of Type 1 Diabetic Mice via Blocking the Aberrant HMGB1/RAGE/NF-κB Pathway

**DOI:** 10.14336/AD.2018.0707

**Published:** 2019-06-01

**Authors:** Rongrong Han, Zeyue Liu, Nannan Sun, Shu Liu, Lanlan Li, Yan Shen, Jianbo Xiu, Qi Xu

**Affiliations:** ^1^State Key Laboratory of Medical Molecular Biology, Institute of Basic Medical Sciences, Chinese Academy of Medical Sciences, School of Basic Medicine Peking Union Medical College, Beijing, China; ^2^Neuroscience center, Chinese Academy of Medical Sciences, Beijing, China

**Keywords:** diabetes, BDNF, neuroinflammation, HMGB1, RAGE

## Abstract

Diabetes is a systemic disease that can cause brain damage such as synaptic impairments in the hippocampus, which is partly because of neuroinflammation induced by hyperglycemia. Brain-derived neurotrophic factor (BDNF) is essential in modulating neuroplasticity. Its role in anti-inflammation in diabetes is largely unknown. In the present study, we investigated the effects of BDNF overexpression on reducing neuroinflammation and the underlying mechanism in mice with type 1 diabetes induced by streptozotocin (STZ). Animals were stereotactically microinjected in the hippocampus with recombinant adeno-associated virus (AAV) expressing BDNF or EGFP. After virus infection, four groups of mice, the EGFP+STZ, BDNF+STZ, EGFP Control and BDNF Control groups, received STZ or vehicle treatment as indicated. Three weeks later brain tissues were collected. We found that BDNF overexpression in the hippocampus significantly rescued STZ-induced decreases in mRNA and protein expression of two synaptic plasticity markers, spinophilin and synaptophysin. More interestingly, BDNF inhibited hyperglycemia-induced microglial activation and reduced elevated levels of inflammatory factors (TNF-α, IL-6). BDNF blocked the increase in HMGB1 levels and specifically, in levels of one of the HMGB1 receptors, RAGE. Downstream of HMGB1/RAGE, the increase in the protein level of phosphorylated NF-κB was also reversed by BDNF in STZ-treated mice. These results show that BDNF overexpression reduces neuroinflammation in the hippocampus of type 1 diabetic mice and suggest that the HMGB1/RAGE/NF-κB signaling pathway may contribute to alleviation of neuroinflammation by BDNF in diabetic mice.

Original Article

Diabetes is one of the most prevalent devastating chronic diseases with the number of affected people predicted to reach 552 million by 2035 [1]. Diabetes is a systemic disease that can damage multiple organs in the body. Complications associated with diabetes include pathologic changes in both the small and large vessels, cranial and peripheral nerves, kidneys, and eyes. Diabetes also increases the risk of cognitive impairment [2] and precipitates Alzheimer’s disease and vascular dementia [3]. Recently, great efforts have been made to investigate the effects of diabetes on the brain, mostly focusing on the synaptic impairments in the hippocampus that may lead to dysfunction in plasticity.[4]. Hyperglycemia triggers a number of mechanisms thought to underlie diabetic neuropathy [5], including oxidative stress, neuro-inflammation, and autophagy [6]. Neuro-inflammation mainly manifests as microglial activation and the increased release of inflammatory factors, such as tumor necrosis factor (TNF) and interleukins (ILs), can directly result in synaptic impairments [7].

Brain-derived neurotrophic factor (BDNF) is a member of the neurotrophin family of growth factors and is essential in modulating neuroplasticity by regulating cell survival, proliferation, and synaptic growth in the developing and adult brain [8, 9]. Recently, it was reported that low expression levels of BDNF are associated with cognitive deficits in patients with diabetes [10]. The expression level of BDNF is significantly decreased in the hippocampus of both type 1 and type 2 diabetes rats [11, 12]. To date, however, whether BDNF is sufficient to mitigate synaptic impairments and reduce neuroinflammation in the diabetic brain has not been investigated. If BDNF is sufficient, what is the underlying mechanism behind its therapeutic effects?

High-mobility group box 1 (HMGB1), a member of the HMG family, is a nonhistone-binding protein and is a potent inflammatory mediator after trauma and injury [13, 14]. Currently three receptors have been identified that transduce the signal from HMGB1, the receptor for advanced glycation end products (RAGE) and Toll-like receptors (TLRs) TLR-2 and TLR-4. The downstream signaling results in the expressions of inflammatory cytokines, chemokines, and corresponding receptors [15]. Some studies have described differential effects of HMGB1-dependent signaling on hyperglycemia-induced complications [16, 17]. Increased HMGB1 expression in the dorsal root ganglion (DRG) is critical for the development of peripheral neuropathy in type 1 diabetes [18]. Activation of HMGB1 receptors can ultimately result in the activation of nuclear factor-kappaB (NF-κB), which may promote inflammation [19]. However, the signaling pathway downstream of HMGB1 that may play a role in hyperglycemia-induced synaptic impairments and neuroinflammation is not yet clear.

In the present study, we aimed to investigate the effects of BDNF overexpression in the hippocampus on mitigating synaptic impairments and reducing neuroinflammation in mice with diabetes induced by STZ. The underlying mechanism related to the HMGB1/ NF-κB signaling pathway was also studied.

## MATERIALS AND METHODS

### Animals

All animal studies were approved by the institutional review board (IRB) of the Institute of Basic Medical Sciences, Chinese Academy of Medical Sciences. Male C57BL/6J mice at the age of eight weeks were purchased from Vital River Laboratories (Vital River Laboratories, Beijing, China). Mice were acclimatized for two weeks before the experiment in a room with a 12-hour light/dark cycle and had unlimited access to food and water. The temperature and humidity were maintained at 23°C ± 2°C and 60% ± 5%, respectively. All mice were handled in accordance with the terms of the Beijing Administration Office of Laboratory Animal Guidelines for the Care and Use of Laboratory Animals.

### Recombinant adeno-associated virus (AAV) construction and packaging

The AAV plasmid used in this study contains a vector expression cassette consisting of one cytomegalovirus (CMV) enhancer fused to the chicken beta-actin (CBA) promoter, one woodchuck posttranscriptional regulatory element (WPRE) and bovine growth hormone polyadenosine flanked by AAV inverted terminal repeats. The mouse BDNF cDNA and the EGFP sequence were linked by a T2A signal to achieve autocleavage after translation. Finally, the BDNF-2A-EGFP sequence was inserted into the multiple cloning sites between the CBA promoter and the WPRE site. The AAV vector that only expressed EGFP was used as control. AAV2/9-BDNF and AAV2/9-GFP were packaged (Taitool Bioscience, China) and the final titers for use were 3.1×10^12^ and 3.3×10^12^ vector genome (v.g.)/ml, respectively.

### AAV vector injection

We randomly divided 60 mice into two groups that received AAV-BDNF (n=30) or AAV-GFP (n=30) injection. After anesthetization with 70 mg/kg of pentobarbital sodium, mice were stereotactically microinjected with 1 µl per side of AAV vector bilaterally into the hippocampus (2.2 mm posterior to the bregma, 2.2 mm lateral to the midline, 1.8 mm dorsal to the bregma) at a rate of 300 nanoliters/min through a Micro4 Micro Syringe Pump Controller (World Precision Instruments, USA).

### Induction of Type 1 Diabetes

Three weeks after virus injection, mice were divided into four experimental groups, namely, BDNF Control (n=14), BDNF+STZ (n=16), EGFP Control (n=14), EGFP+STZ (n=16). Type 1 diabetes was induced by 200 mg/kg of streptozotocin (STZ; Sigma, USA) that was dissolved in a citrate buffer (pH 4.3) and administered via intraperitoneal injection (i.p.). Mice in the control group received vehicle alone. Two days after injection, the mouse tail was pierced by a needle and a drop of venous blood was taken to measure blood glucose. Blood glucose was determined by a glucometer (Roche Diagnostics, Switzerland) and only animals with blood glucose higher than 16.7 mmol/L were considered diabetic and included in the study. Body weight and blood glucose were recorded at the beginning and every week after STZ injection for the duration of the experiment.

### Western blot analysis

Mice were sacrificed by decapitation under deep anesthesia with chloral hydrate (10%, wt/vol, i.p.). The hippocampus was rapidly dissected and frozen. For Western blot analysis, the hippocampus of one hemisphere was lysed by ultrasonication in lysis buffer (150 mM NaCl, 50 mM Tris-HCl (pH=7.5), 0.1% sodium deoxycholate, 1% Triton-X-100, 0.1% SDS, 1% Protease Inhibitor Cocktail and Phosphatase Inhibitor (Roche, Switzerland)) and centrifuged. Protein concentrations were determined using the bicinchoninic acid (BCA) method (Thermo Fisher Scientific, USA). Equal quantities (20 µg) of tissue extracts were separated by electrophoresis through 12% polyacrylamide gel and then transferred to NC membranes (Millipore, Germany). The membrane was then incubated for 60 min in blocking buffer (TBST buffer containing 5% skim milk powder) at room temperature. Next, the membranes were incubated overnight at 4°C with primary antibodies. Then, the membranes were incubated with secondary antibodies for 60 min at room temperature. The specific reaction was visualized by a chemiluminescence substrate luminol reagent (Promega). The optical density of protein was analyzed by densitometric measurements using Image-Pro Plus software 5.0 (Rockville, USA) according to the manufacturer’s instructions. The following primary antibodies were used: anti-HMGB1 (1:5000, Abcam, USA), anti-RAGE (1:1000, Abcam, USA), anti-spinophilin (1:1000, Abcam, USA), anti-synaptophysin (1:1000, Sigma, USA), anti-BDNF (1:200, Alomone labs, Israel), anti- NF-κB p65(1:1000, Cell Signaling Technology, USA), anti-p-TrkB (1:400, Abcam, USA), anti-GAPDH (1:1000, Cell Signaling Technology, USA), anti-actin (1:2000, Sigma, USA),anti-tubulin(1:1000, Cell Signaling Technology, USA). Data were normalized to GAPDH, actin or tubulin immunolabeling and expressed as percentage of control values.

### Quantitative real-time PCR

Total RNA from the hippocampus of the other hemisphere was extracted using TRIzol (Thermo Fisher Scientific, USA) according to the manufacturer’s protocol and samples were stored at -80°C. The cDNA was synthesized using a Fast Lane Cell cDNA kit (QIAGEN, Germany). Relative gene expression was assessed by real-time PCR using FastStart Universal SYBR Green Master Mix (Roche, Switzerland). A fragment of GAPDH was amplified as the internal control. The primers used in this study are shown in table 1. Differences in gene expression were calculated using the 2^-ΔΔCT^ method and are presented as the relative fold change.

**Table 1:** Primers used in the study.

Gene names	Primer sequences
HMGB1	Forward: 5′-TGCCTCGCGGAGGAAAATC-3′
	Reverse: 5′-AACGAGCCTTGTCAGCCTTT-3′
RAGE	Forward: 5′-GAATAGTCGCTCCTGGTGGG-3′
	Reverse: 5′-CAGCTATAGGTGCCCTCATCC-3′
TLR2	Forward: 5′- GTGCGGACTGTTTCCTTCTG-3′
	Reverse: 5′- GCGTTTGCTGAAGAGGACTG-3′
TLR4	Forward: 5′-ATCCCTGCATAGAGGTAGTTCC-3′
	Reverse: 5′-GGTGGTGTAAGCCATGCCA-3′
Synaptophysin	Forward: 5′- TCGTGTTCAAGGAGACAGGC -3′
	Reverse: 5′- CTGCCCGTAATCGGGTTGAT -3′
Spinophilin	Forward: 5′- AGGGAGGGAGGTCAGCTTAG -3′
	Reverse: 5′- AAACGTTGGAAAACAGCGGG -3′
GAPDH	Forward: 5′- CCTGCTTCACCACCTTCTTGA-3′
	Reverse: 5′- TGTGTCCGTCGTGGATCTGA-3′

### Immunofluorescence and quantification procedures

For immunostaining, mice were rapidly anesthetized with chloral hydrate (10%, wt/vol, i.p.) and sequentially perfused transcardially with ice-cold 0.01 M phosphate-buffered saline (PBS, pH 7.4) and paraformaldehyde (4% in PBS, wt/vol, Sigma, USA). Brains were postfixed for 24 h in the same fixative solution and stored at 4°C. After dehydration with 30% sucrose (wt/vol) in 0.01 M PBS for 24 hours, three serial 40-μm-thick coronal sections were cut on a cryostat (CM1950, Leica, Germany) and immediately subjected to immunofluorescent staining. The following primary antibodies were used: anti-HMGB1 antibody (1:400, Abcam, USA) and anti-Iba1 antibody (1:400, Abcam, USA). After incubation with the mixed primary antibodies at 4 °C overnight, slices for immunofluorescence assays were incubated with the corresponding secondary antibody conjugated with Alexa 594 (1:1000, Invitrogen, USA) overnight at 4 °C. All images were captured by an LSM780 confocal laser scanning microscope (Zeiss, Germany) using identical camera acquisition settings for each marker. Images shown in the figures were from the same region illustrated in the schematics according to specific neuroanatomical markers.

Quantification of brain sections was performed using ImageJ software. The expression of HMGB1 in four mice from each group was quantified by measuring the average fluorescence intensity of the whole hippocampus area. For microglia quantification, three to four representative images from one mouse and a total of four mice from each group were analyzed. A threshold for positive staining was determined for each image that included all cell bodies and processes but excluded background staining. The results were shown as the average percent area in the positive threshold for all representative pictures [20].

### Hippocampal TNF-α, IL-6 and IL-1β levels

The hippocampus was dissected from the brain, homogenized in PBS buffer containing 1% Protease Inhibitor Cocktail and Phosphatase Inhibitor (Roche, Switzerland), sonicated, and centrifuged at 12,000 rpm for 15 min at 4°C. The supernatant fraction was separated and used for further study. Commercially available kits from R&D Systems (USA) were used to determine the concentrations of TNF-α, IL-6 and IL-1β. The procedure for ELISA was performed according to the manufacturer’s instructions. The level of the total protein in the supernatant was estimated and levels of TNF-α, IL-6 and IL-1β were expressed as picograms per milligram of total protein.

### Data analysis

Statistical significance was calculated using Prism software (GraphPad 6.0, USA). Before calculation, all data were tested with normal distribution analysis. In comparing the difference between two groups, unpaired two-sided Student’s t test was used. One-way ANOVA followed by Tukey’s or Dunnett’s tests were used for multiple comparisons. The data were presented as the mean ± standard error of the mean (SEM) unless otherwise noted, with *P* < 0.05 as the criterion for statistical significance. All animals were present at the end of the study. No data points were excluded. All histopathological analyses were performed in a blinded manner.

**Figure 1.**
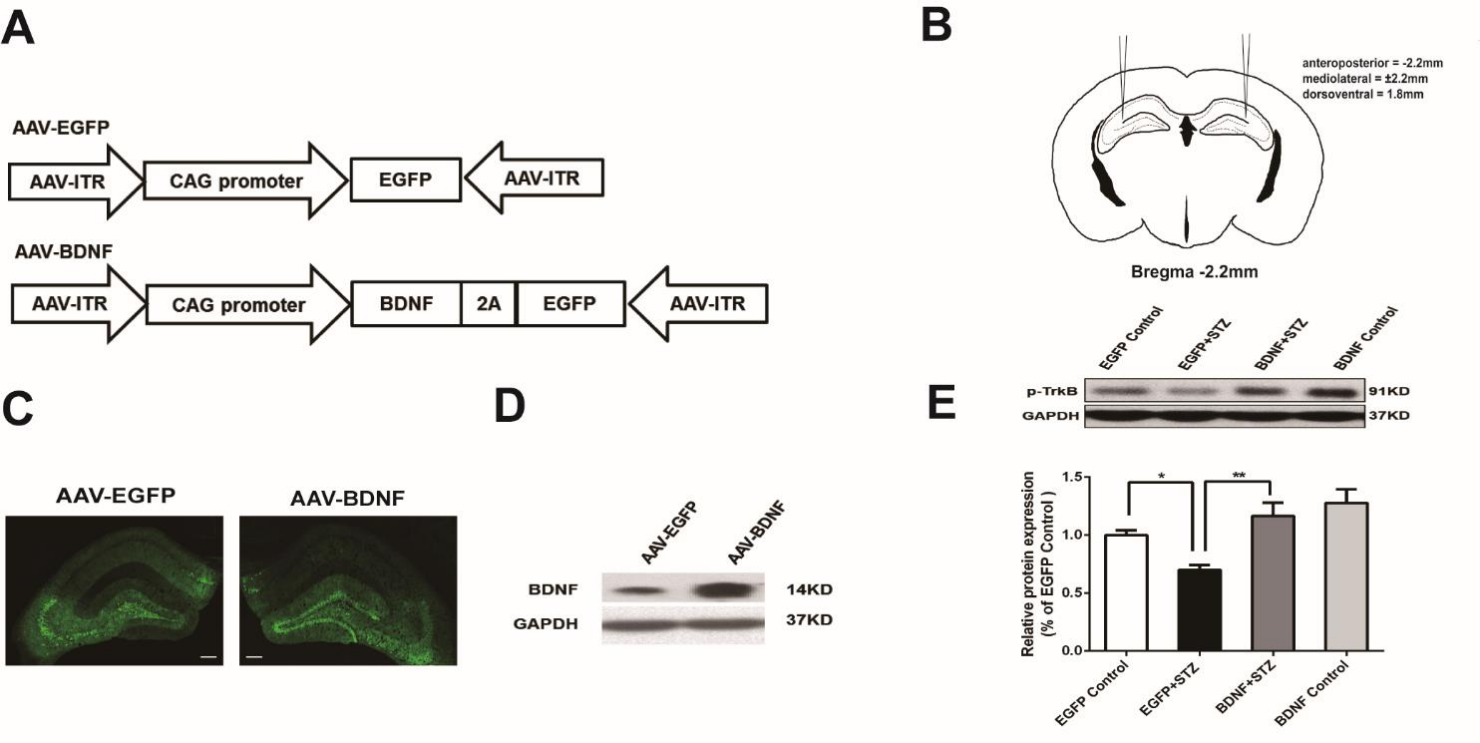
BDNF gene transfer in the hippocampus (**A**) Schematics of the AAV vector construction. (**B**) Schematics illustrating virus microinjection. (**C**) EGFP fluorescence demonstrating the site of virus expression. Scale bars represent 200 μm. (**D**) Western blot analysis of BDNF expression in the hippocampus after virus injection. (**E**) Western blot assay of p-TrkB. Values are means ± SEM. n = 6-7 per group. * p < 0.05, ** p < 0.01.

## RESULTS

### BDNF gene transfer in the hippocampus

To overexpress BDNF in the hippocampus, we utilized AAV as the vector for in vivo gene transfer. Currently, AAV is the most potent tool for gene therapy owing to its persistent existence as an episome and low toxicity. First, we constructed the recombinant AAV vector that expresses mouse BDNF and EGFP simultaneously under the control of an autocleavage peptide T2A (Fig. 1A). The AAV vector that only expressed EGFP was used as control. After the virus was packaged, we stereotactically microinjected 1 μl per side of AAV-EGFP or AAV-BDNF bilaterally into the hippocampus (Fig. 1B). In a subset of mice, the efficacy of the AAV-mediated gene transfer was determined by measuring the EGFP fluorescence. Both AAV-EGFP and AAV-BDNF groups showed high expression of EGFP in the hippocampus, mainly distributed in the dentate gyrus and the CA3 area (Fig. 1C). The Western blot assay further demonstrated that the expression level of the BDNF protein in the AAV-BDNF group was markedly higher than that in the AAV-EGFP group (Fig. 1D). It is well known that BDNF binds to tropomyosin-related kinase receptor B (TrkB), induces autophosphorylation of TrkB, and then triggers several signaling cascades that affect multiple cellular processes [21]. The expression level of BDNF is significantly decreased in the hippocampus of both type 1 and type 2 diabetes rats [11, 12]. Therefore, we examined the level of phosphorylated TrkB after BDNF overexpression in the hippocampus. As expected, the phosphorylation of TrkB was significantly decreased in the EGFP+STZ group compared with that in the EGFP Control group (p<0.05), while it significantly increased in the BDNF+STZ group compared with the EGFP+STZ group (p<0.01) (Fig. 1E). These results indicated that BDNF was successfully overexpressed in the hippocampus of mice and may have functional effects through activating the downstream signaling pathways.

**Figure 2.**
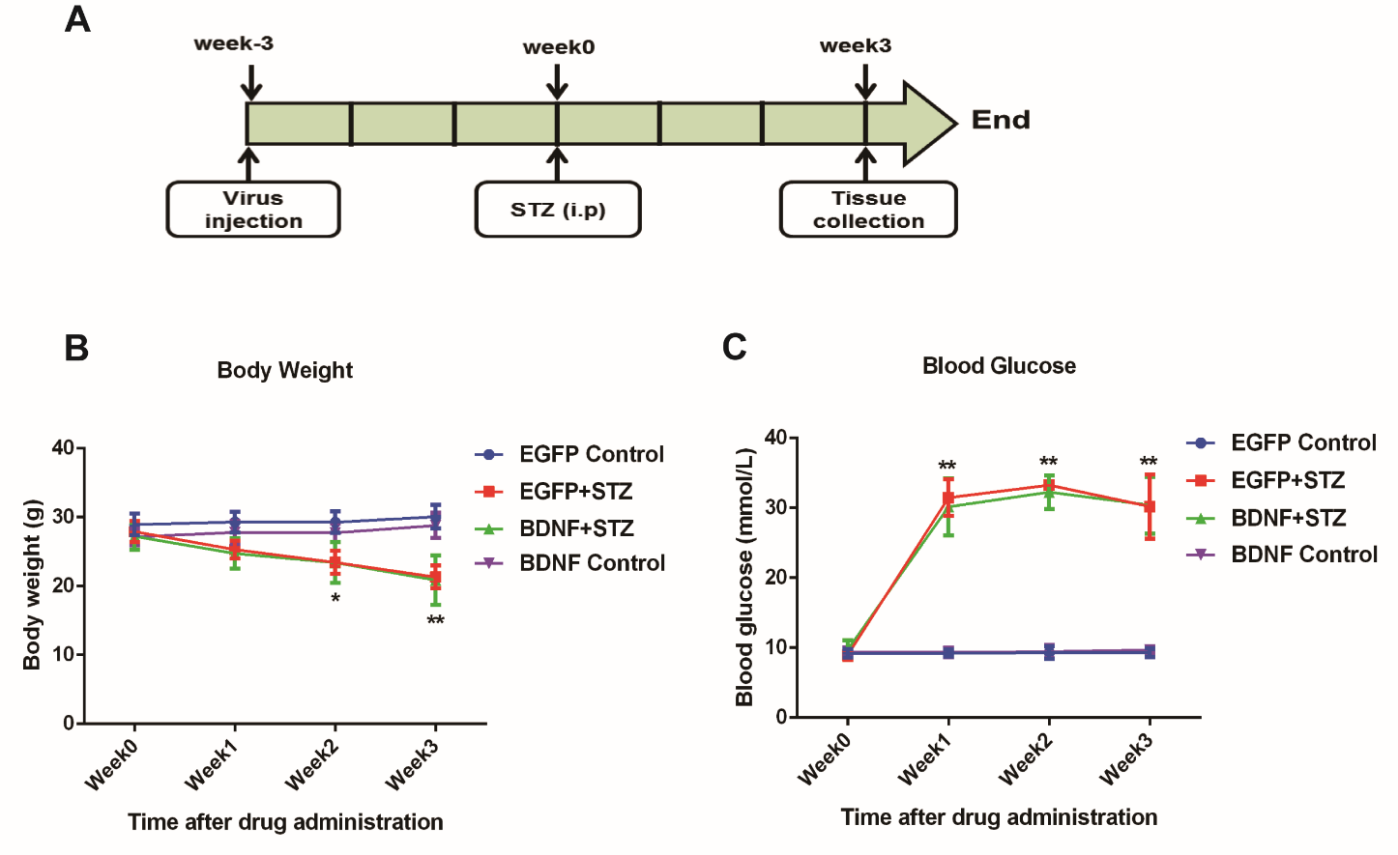
STZ-induced type 1 diabetes in mice (**A**) Schematic representation of the study design and treatment schedule. (**B**) Body weight after STZ treatment. (**C**) Blood glucose levels after STZ treatment. Values are means ± SEM. n = 10-12 per group. * p < 0.05, ** p < 0.01, indicating the comparison between the EGFP Control and EGFP+STZ groups.

### STZ-induced type 1 diabetes in mice

Three weeks after virus administration, STZ was used to induce type 1 diabetes in mice via one single intraperitoneal injection at a dose of 200 mg/kg, and thereafter body weight and blood glucose were measured weekly before sacrificing animals to collect tissues (Fig. 2A). After STZ administration, the body weight of mice in the EGFP+STZ and BDNF+STZ groups decreased every week and were significantly lower than that of the mice in the EGFP Control and BDNF Control groups on the third week (p<0.01). There was no difference in body weight between the mice in the EGFP+STZ and BDNF+STZ groups (Fig. 2B). The level of blood glucose of mice in the EGFP+STZ and BDNF+STZ groups became significantly higher than that of mice in the EGFP Control and BDNF Control groups in the first week (p<0.01) and remained at the same level over the following two weeks. There was also no difference in blood glucose level between the mice in the EGFP+STZ and BDNF+STZ groups (Fig. 2C). The results showed that type 1 diabetes was successfully developed in mice, and BDNF overexpression in the hippocampus had no effects on relieving hyperglycemia induced by STZ.

**Figure 3.**
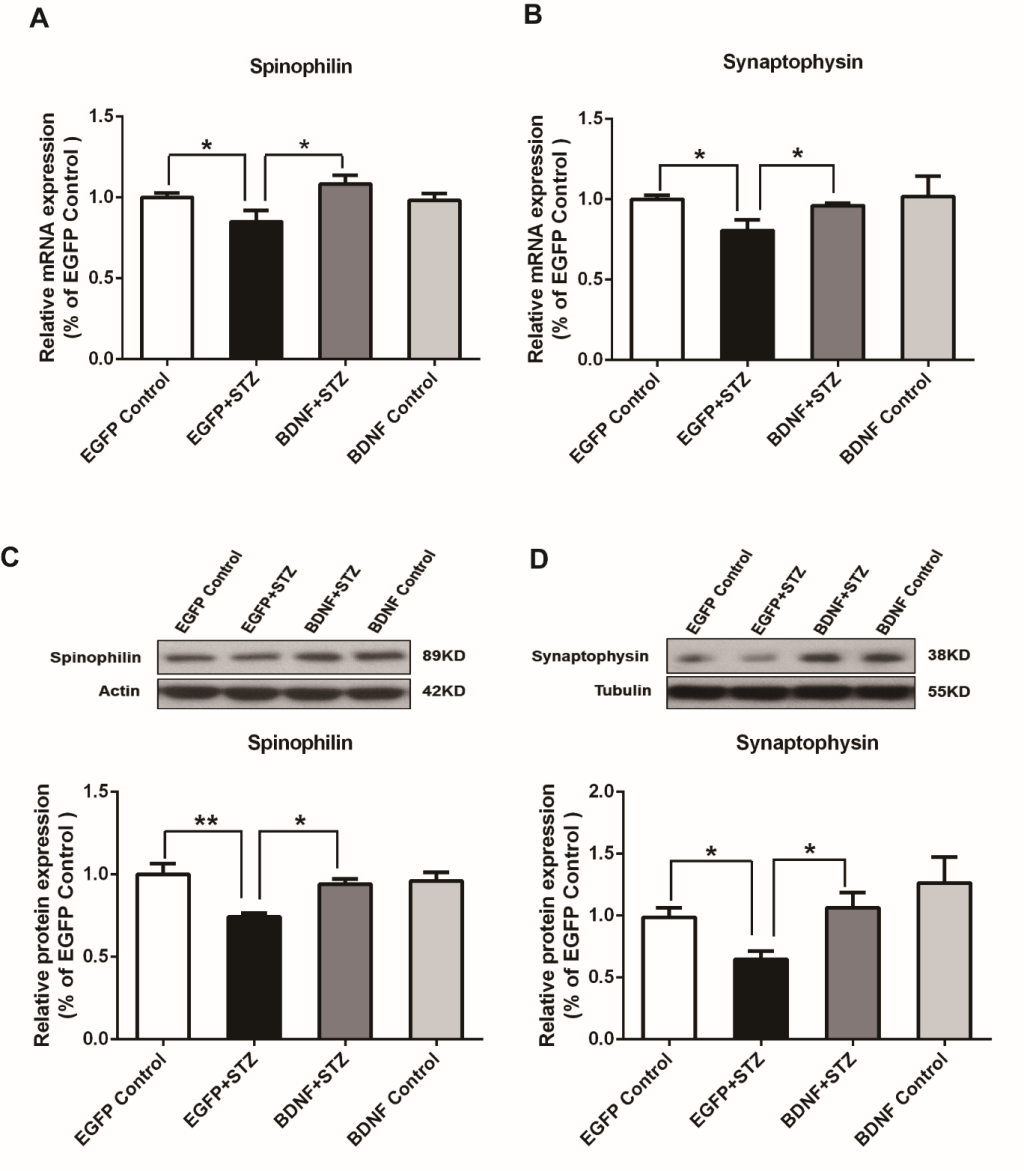
BDNF blocked the hyperglycemia-induced decrease in the expression of spinophilin and synaptophysin of diabetic mice (A) Real time PCR analysis of spinophilin. (B) Real time PCR analysis of synaptophysin. (C) Western blot assay of spinophilin. (D) Western blot assay of synaptophysin. Values are means ± SEM. n = 6-7 per group. * p < 0.05, ** p < 0.01.

**Figure 4.**
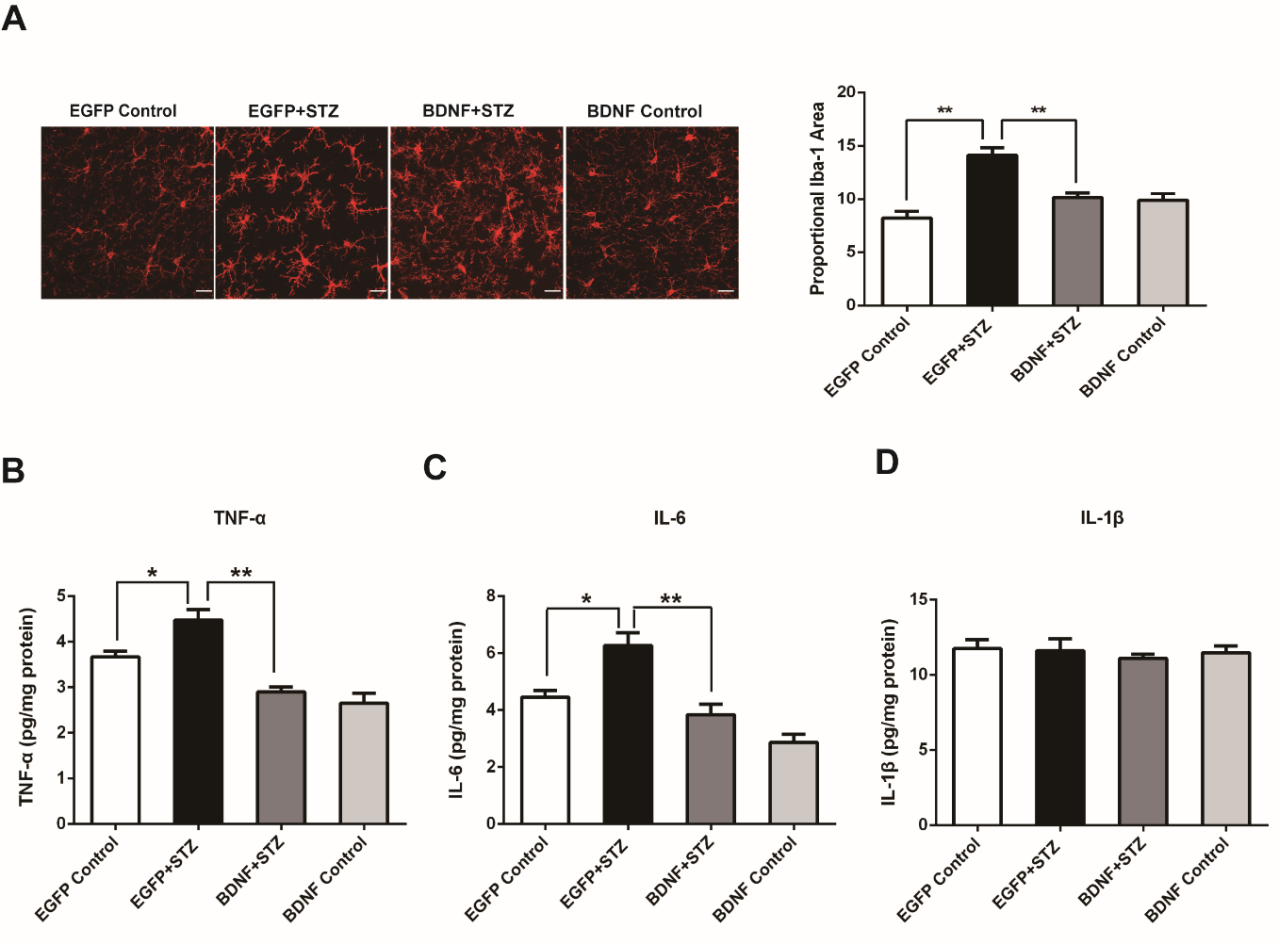
BDNF overexpression suppressed microglial activation and reduced the increased levels of TNF-α and IL-6 in the hippocampus of diabetic mice (A) Immunofluorescent staining and quantification of the microglial marker Iba-1. (B) ELISA assay of TNF-α. (C) ELISA assay of IL-6. (D) ELISA assay of IL-1β. Values are means ± SEM. n = 6-7 per group. * p < 0.05, ** p < 0.01. Scale bars = 20 μm.

### BDNF blocked the hyperglycemia-induced decrease in the expression of spinophilin and synaptophysin

Hyperglycemia has been reported to impair a variety of neurophysiological functions including synaptic transmission [22]. Owing to its various roles in modulating synaptic functions, a BDNF-based synaptic repair strategy has been proposed to treat neuro-degenerative diseases [23]. Therefore, in the STZ-induced diabetic mice we examined the expression of two synaptic protein markers, spinophilin and synaptophysin, which are located post-and presynaptically respectively [24, 25]. Spinophilin can be recruited to the synapse in response to *N*-methyl-D-aspartate (NMDA) and α-amino-3-hydroxy-5-methyl-4-isoxazolepropionic acid receptor (AMPAR) activation to maintain long term depression [26]. Synaptophysin is the first synaptic vesicle protein to be cloned and characterized and regulates endocytosis to ensure vesicle availability during and after sustained neuronal activity [27]. Both proteins are involved in the normal functions of the synapse. Real-time PCR analysis revealed that the expression of both spinophilin and synaptophysin mRNAs was significantly decreased in the EGFP+STZ group (p<0.05) compared with that in the EGFP Control group, while it was significantly increased (p<0.05) in the BDNF+STZ group compared with that in the EGFP+STZ group. There was no difference in expression between the EGFP Control and BDNF Control groups (Fig. 3A & 3B). Western blot analysis yielded similar results. BDNF significantly increased the expression of both spinophilin and synaptophysin proteins in the hippocampus of STZ-treated mice compared with that in the EGFP+STZ mice (p<0.05), while there was no difference between the BDNF Control and EGFP Control mice (Fig. 3C & 3D). These results suggest that BDNF overexpression may reverse the hyperglycemia-induced synaptic impairments in the hippocampus via blocking the decrease in expression of synaptic plasticity-related proteins.

**Figure 5.**
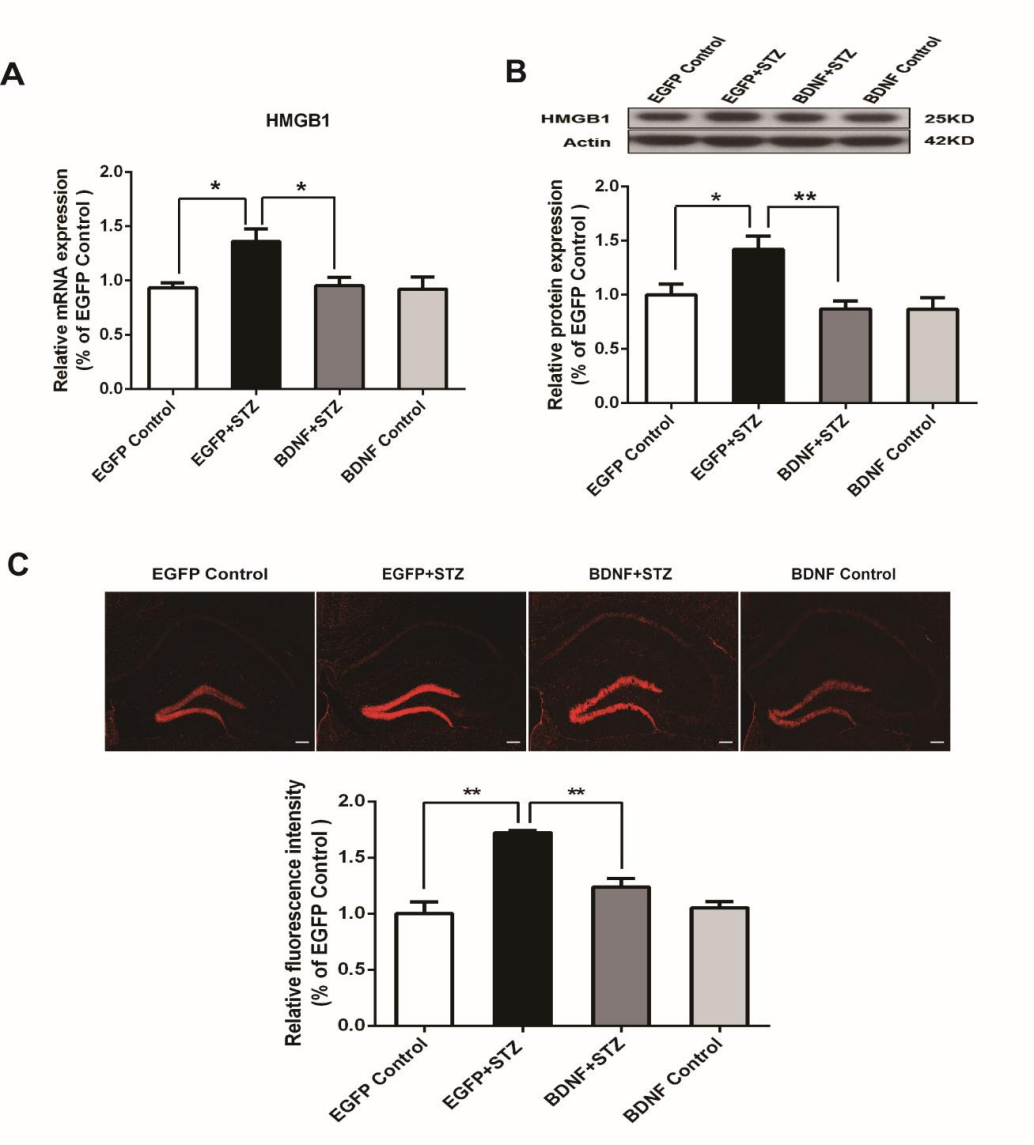
BDNF suppressed the increased expression of HMGB1 in the hippocampus of diabetic mice (A) Real time PCR analysis of HMGB1. (B) Western blot assay of HMGB1. (C) Immunofluorescent staining and quantification of HMGB1. The values are expressed as mean ± SEM. n = 6-7 per group. * p < 0.05, ** p < 0.01. Scale bars = 200 μm.

### BDNF overexpression suppressed microglial activation and reduced the increased levels of TNF-α and IL-6 in the hippocampus of diabetic mice

Considering the serious consequences of microglial activation and neuroinflammation on synaptic functions [28-30], the effects of BDNF overexpression on microglial activation and levels of inflammatory factors in hippocampus were investigated. Immunofluorescent staining for the ionized calcium-binding adaptor molecule 1 (Iba-1), which is a marker for microglia demonstrated that the microglia showed smaller, round soma with ramified processes in the EGFP Control group, which is the typical morphology of resting microglia. However, in the EGFP+STZ group, the microglia showed much larger somal diameters and increased varicosities in their process fields, which is indicative of microglial activation. Iba-1 immunoreactivity of microglia was significantly increased in the EGFP+STZ group compared with that in the EGFP Control group, (p<0.01), while BDNF significantly blocked the increase in Iba-1 immunoreactivity compared with that in the EGFP+STZ group (p<0.01). These results indicated that BDNF markedly inhibited microglial activation in the hippocampus of diabetic mice (Fig. 4A). The concentrations of inflammatory factors including TNF-α, IL-1β and IL-6 were measured by ELISA. The results showed that the EGFP+STZ group had significantly higher expression levels of TNF-α and IL-6 compared with those of the EGFP control group (p<0.05), while BDNF significantly blocked the increased levels of TNF-α and IL-6 in the STZ-treated mice (p<0.01) (Fig. 4B & 4C). The level of IL-1β showed no difference among the four groups. These results suggest that BDNF overexpression may ameliorate neuroinflammation in the hippocampus of diabetic mice.

**Figure 6.**
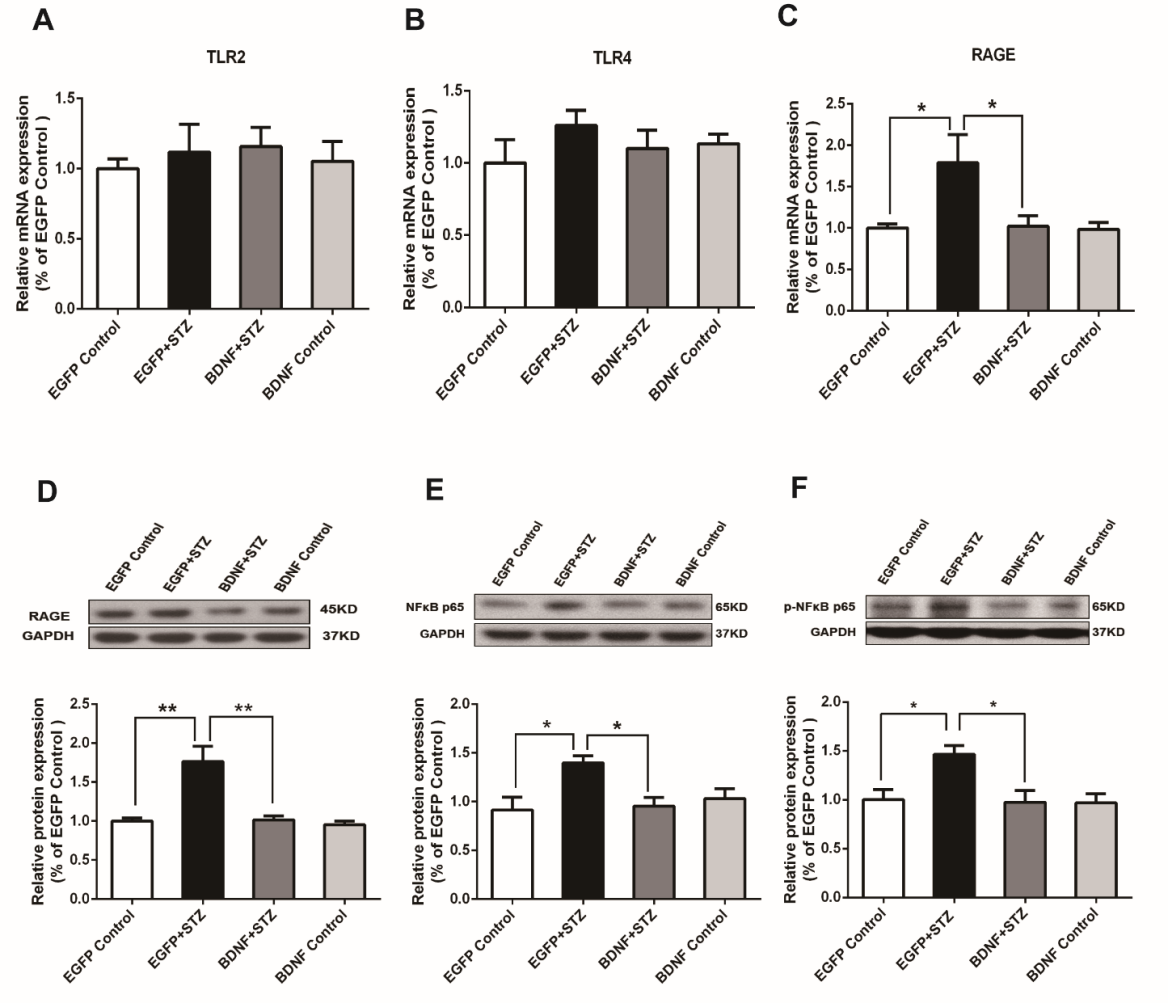
BDNF inhibited HMGB1-mediated activation of the RAGE/NF-κB signaling pathway in the hippocampus of diabetic mice (A) Real time PCR analysis of the HMGB1 receptor TLR2. (B) Real time PCR analysis of the HMGB1 receptor TLR4. (C) Real time PCR analysis of the HMGB1 receptor RAGE. (D) Western blot assay of RAGE. (E) Western blot assay of NF-κB p65. (F) Western blot assay of p-NF-κB p65. The values are expressed as means ± SEM. n = 6-7 in each group. * p < 0.05, ** p < 0.01.

### BDNF suppressed the increased expression of HMGB1 in the hippocampus of diabetic mice

As HMGB1 played an important role in microglial activation and the subsequent inflammatory response [31, 32], we studied whether BDNF had any effect on the expression of HMGB1. The results of the real-time PCR assay demonstrated that mRNA expression of HMGB1 in the EGFP+STZ group was significantly increased (p<0.05) compared with that in the EGFP Control group, but BDNF effectively suppressed the expression of HMGB1 in the BDNF+STZ group (p<0.05) compared with that in the EGFP+STZ group (Fig. 5A). Western blot analysis also showed significantly decreased expression of HMGB1 in the BDNF+STZ group compared with that in the EGFP+STZ group (p<0.01) (Fig. 5B), and immunofluorescent staining of HMGB1 further confirmed the results (Fig. 5C). These results suggest that the anti-inflammatory effects of BDNF may be exerted through suppression of the expression of HMGB1 in the hippocampus of diabetic mice.

### BDNF inhibited HMGB1-mediated activation of the RAGE/NF-κB signaling pathway in the hippocampus of diabetic mice

Currently three receptors, RAGE, TLR-2 and TLR-4, have been identified that transduce the downstream signaling of HMGB1 [15]. We further characterized which specific pathway mediates the signal downstream of HMGB1 in the hippocampus of diabetic mice. Changes in the expression of these receptors were measured using real-time PCR. The expression levels of TLR2 and TLR4 showed no differences among the four groups (Fig. 6A & 6B). Only the expression of RAGE mRNA was significantly increased in the EGFP+STZ group (p<0.05) compared with that in the EGFP Control group and was significantly inhibited by BDNF overexpression in the BDNF+STZ group (p<0.05) compared with expression in the EGFP+STZ group (Fig. 6C). The Western blot assay further confirmed the results showing that the protein level of RAGE was significantly suppressed in the BDNF+STZ group (p<0.01) compared with that in the EGFP+STZ group (Fig. 6D). The RAGE-dependent activation of NF-κB has been implicated in diabetes previously [33]. Therefore, the expression levels of NF-κB and phosphorylated NF-κB (p-NF-κB) were also detected. We found that hyperglycemia significantly increased the expression of NF-κB p65 and p-NF-κB p65 in the hippocampus (p<0.05), while BDNF significantly decreased the expressions (p<0.05) compared with that in the EGFP+STZ group (Fig. 6E & 6F). These results suggest that the HMGB1/RAGE/NF-κB pathway may be specifically involved in the neuroinflammatory pathogenesis in the hippocampus of diabetic mice and that the anti-inflammatory effects of BDNF may be exerted through the suppression of this signaling pathway.

## DISCUSSION

**Figure 7.**
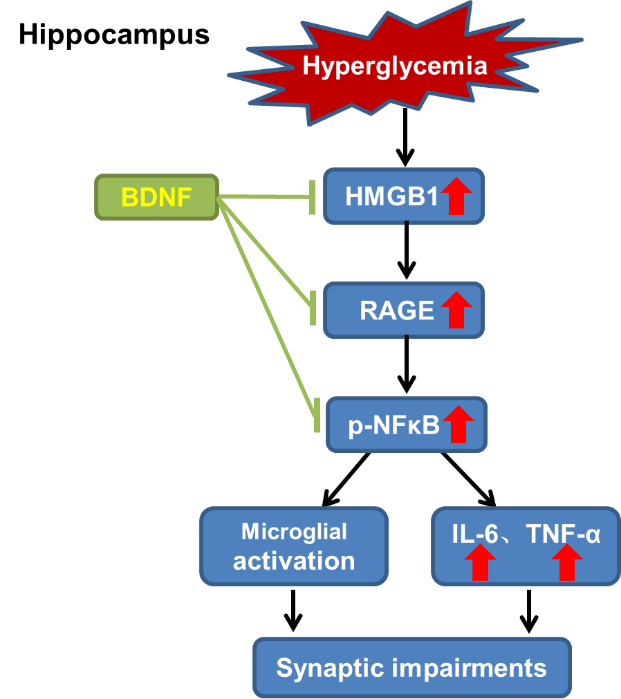
Schematics illustrating the possible neuroprotective mechanisms of BDNF in the hyperglycemia-induced neuroinflammation In the hippocampus of the diabetic brain, hyperglycemia leads to microglial activation and increased levels of inflammatory factors, ultimately resulting in synaptic impairments. BDNF can alleviate the hyperglycemia-induced neuroinflammation via specifically inhibiting the aberrant HMGB1/ RAGE/NF-κB signaling pathway.

In the present study, we have shown that hyperglycemia causes a decrease in synaptic marker expression and neuroinflammation in the hippocampus. BDNF overexpression mitigates the synaptic impairments and suppresses the microglial activation and elevated levels of inflammatory factors that may be mediated by specifically inhibiting the HMGB1/RAGE/NF-κB signaling pathway in the hippocampus of diabetic mice. In the STZ-induced T1D mouse model, we found that overexpression of BDNF in the hippocampus can rescue the decreased expression of spinophilin and synaptophysin, two synapse-associated proteins. BDNF also restores the morphology of activated microglia induced by hyperglycemia close to the resting state and suppresses the increased levels of TNF-α and IL-6. We also observed that the HMGB1/RAGE/NF-κB signaling pathway is specifically activated in the hippocampus of diabetic mice, which may lead to microglial activation and neuroinflammation, ultimately resulting in synaptic impairments (Fig. 7).

Severe hyperglycemia can induce permanent neurologic sequelae including neuronal cell death [34], blood-brain barrier disruption [35], and abnormalities in synaptic transmission [36], which may accelerate cognitive impairment. BDNF plays an essential role in regulating synaptic plasticity [37], which is thought to be the cellular mechanism for learning and memory in adult brains [38]. Previous studies have found reduced serum levels of BDNF in patients with type 2 diabetes [10]. Moreover, high levels of glucose inhibit the output of BDNF from the human brain [39]. Expression of BDNF mRNA and protein were also reported to be markedly decreased in the hippocampus of *db/db* mice and STZ-induced diabetic rats [40, 41]. However, whether BDNF is sufficient to prevent or reverse the hyperglycemia-induced brain damages has not been directly investigated. To our knowledge, this is the first study showing that BDNF overexpression in the hippocampus of STZ-induced diabetic mice prevents the decreased expression of two synapse-associated proteins, spinophilin and synaptophysin that are important for normal synaptic function (Fig. 3), suggesting a neuroprotective role of BDNF in the hippocampus of diabetic brains. It should be noted that the hippocampal overexpression of BDNF has no effects on ameliorating hyperglycemia (Fig. 2), although BDNF action via i.c.v. infusion [42] or in the hypothalamus via AAV-mediated gene transfer [43] substantially lowers blood glucose levels, suggesting that the antidiabetic role of BDNF is brain region-specific.

Several mechanisms underlying the hyperglycemia-induced brain injury have been proposed, including blood-brain barrier interruption, oxidative stress, mitochondrial dysfunction, apoptosis, reduction of neurotrophic factors, and neuroinflammation that is accompanied by microglial activation and cytokine release [44, 45]. As the resident immune cells in the brain, microglia have an important role in regulating neural plasticity since they assist in the development and maintenance of neuronal networks via trophic factor release and controlled phagocytosis, which allows microglia to prune synapses and remodel neuronal transmission [30, 46]. BDNF released from microglia promotes learning-dependent synapse formation and increases neuronal TrkB phosphorylation, a key mediator of synaptic plasticity [47]. Activation of microglia is a central event and driving force in the pathogenesis of neuroinflammation [48, 49]. Cytokines such as TNF-α, IL-6, and IL-1β can induce proinflammatory responses in the central nervous system resulting in neuroinflammation [50]. In our study, microglia were typically activated, and levels of TNF-α and IL-6 were significantly increased in the hippocampus of diabetic mice; however, BDNF overexpression alleviated this neuroinflammatory damage (Fig. 4). This shows that BDNF may exert an anti-inflammatory role in the diabetic hippocampus in addition to modulating synaptic function. Previous studies have observed the positive correlation between attenuated neuroinflammation and increased BDNF levels, which is presumed to be a possible mechanism underlying treatments for many neurological diseases such as multiple sclerosis [51], Parkinson’s disease [52], cognitive impairment [53-55], and mood disorders [56-58]. Direct evidence comes from a study that combined overexpression of FGF-2 and BDNF in the hippocampus to attenuate epileptogenesis-associated neuro-inflammation and reduces spontaneous recurrent seizures [59]. One recent study also revealed that i.c.v. infusion of BDNF into the brain reduces inflammation and hippocampal apoptosis in experimental *Streptococcus pneumoniae meningitis* [60]. However, whether BDNF can alleviate neuroinflammation induced by hyperglycemia has not been investigated until now. Our results further corroborate the finding that BDNF is sufficient to reduce neuroinflammation, providing another therapeutic target to treat brain injury in diabetes.

What, then are the mechanisms underlying the anti-inflammatory effects of BDNF? This question has just begun to be elucidated. One new study found that BDNF is associated with reducing the release of inflammatory factors from cultured microglia, which may be mediated by the BDNF-EPO-Shh signaling pathway [61]. High-mobility group box 1 (HMGB1), one of the best characterized danger-associated molecular pattern molecules, or alarmins, is an evolutionarily conserved chromosomal protein and is a potent inflammatory mediator after trauma and injury [13, 14]. The actions of HMGB1 in diabetes have been closely investigated since elevated expression levels of HMGB1 have been observed in patients with diabetes [62, 63] and its multiple complications including retinopathy [64], nephropathy [65], cardiomyopathy [66], liver injury [67], and peripheral neuropathy[18]. Functional studies revealed that blockade of HMGB1 may protect against hyperglycemia-induced tissue injury [68-71]. However, whether HMGB1 is involved in the neuroinflammation and the ensuing synaptic dysfunction in the diabetic brain has not been examined. Our study showed that hyperglycemia results in enhanced expression of HMGB1 mRNA and protein, while BDNF overexpression inhibits the increased expression of HMGB1 in the hippocampus of diabetic mice, suggesting a possible role of HMGB1 in mediating the anti-inflammatory effects of BDNF (Fig. 5).

The proinflammatory signaling of HMGB1 is mainly transduced by RAGE and TLRs, among which RAGE was the first receptor identified to bind HMGB1 [72]. The HMGB1/RAGE axis has been suggested to contribute to the pathogenesis of many diseases such as autoimmune diseases [73], cancer [74], and diabetes [75]. Zhang et al. demonstrated that metformin protects against hyperglycemia-induced cardiomyocyte injury by inhibiting the expression of RAGE and HMGB1 [76]. In our results, among the three receptors of HMGB1, only RAGE was upregulated in the hippocampus of STZ-treated mice, suggesting that the HMGB1/RAGE signaling pathway is specifically activated in the diabetic hippocampus. BDNF overexpression inhibited both RAGE mRNA and protein expression in the hippocampus of diabetic mice, suggesting that the BDNF/ HMGB1/RAGE signaling pathway may contribute to the anti-inflammatory effects of BDNF (Fig. 6). Further, in the hippocampus of STZ-treated mice we also detected increased protein expression of phosphorylated NF-κB, which is at the hub of controlling immunity and inflammation, suggesting that activation of the HMGB1/RAGE/NF-κB signaling pathway may be the culprit for hyperglycemia-induced inflammation in the hippocampus. Importantly, BDNF overexpression can inactivate the HMGB1/RAGE/NF-κB signaling pathway via downregulating the expression levels of all three proteins in the hippocampus of diabetic mice. Activation of the HMGB1/RAGE/NF-κB signaling pathway has been reported to be involved in regulating neurite outgrowth [77], HMGB1-mediated chemotaxis [78], autoimmune myocarditis and inflammatory cardiomyopathy [79]. NF-κB activation may lead to microglial activation and an increased release of inflammatory cytokines [80], ultimately impairing synaptic function.

In summary, we found that BDNF overexpression in the hippocampus mitigates synaptic impairments and ameliorates neuroinflammation induced by hyper-glycemia, which may be mediated by inhibiting the HMGB1/RAGE/NF-κB signaling pathway. Therefore, like BDNF, blockage of the HMGB1/RAGE/NF-κB signaling pathway might represent a novel therapeutic strategy in the treatment of diabetic neuropathy. Toward this end, much more work needs to be done, especially with regard to elucidating the detailed ramifications of the HMGB1/RAGE/NF-κB signaling pathway.
